# Exploring the anticancer potential of traditional herbs from Tamil Nadu: a narrative review of ethnomedicinal insights and scientific evidence

**DOI:** 10.3389/fimmu.2025.1680062

**Published:** 2025-11-19

**Authors:** Pradeep M. K. Nair, Ayswarya Rohini Pandian, Vaishali Mathapati, Shobhitha Tantry B., Abhay Sai, Navya Pai, Shanmugam Sudarshan, Manickam Mahalingam, Karishma Silwal

**Affiliations:** 1 Mirakle Integrated Health Centre, Pollachi, India; 2 International Institute of Yoga and Naturopathy Medical Sciences, Chengalpattu, India; 3 Swami Vivekananda Yoga Anusandhana Samsthana, Bengaluru, India; 4 SDM College of Naturopathy and Yogic Sciences, Ujire, India; 5 Sant Hirdaram Medical College of Naturopathy & Yogic Sciences for Women, Bhopal, India

**Keywords:** traditional medicine, anticancer herbs, phytochemicals, integrative oncology, herbal medicine, ethnomedicine

## Abstract

**Background:**

India, has a rich ethnomedicinal tradition where numerous herbs are used in cancer care. However, scientific validation of these practices remains limited. This narrative review explores the phytochemical mechanisms underlying their anti-cancer effects of 32 herbs identified by herbal activists, including physicians and traditional healers from Tamil Nadu, India, for their purported anticancer properties.

**Methods:**

A narrative review was conducted using PubMed, Scopus, and Google Scholar to identify studies published between 2014 and January 2025 on 32 selected anecdotal herbs. Eligible studies included *in vitro*, *in vivo*, clinical, and in silico investigations. Data extraction by five independent reviewers focused on botanical and common names, bioactive compounds, mechanisms of anticancer activity, cancer types studied, and evidence level (preclinical vs. clinical).

**Results:**

Herbs such as *Withania somnifera*, *Curcuma longa*, and *Annona muricata* exhibited strong preclinical and limited clinical anticancer activity through apoptosis induction, inhibition of angiogenesis and metastasis, immune modulation, and synergy with standard therapies. Most other herbs remain at the preclinical stage, with minimal clinical data. Only *Catharanthus roseus* (purified) and *Curcuma longa* (formulations) have limited clinical application. Challenges including poor bioavailability, lack of standardization, safety concerns, and toxicity (e.g., *Annona muricata*, *Gloriosa superba*) hinder clinical translation, underscoring the need for rigorous evaluation.

**Conclusion:**

Traditional herbs demonstrate notable experimental anticancer potential, yet clinical validation is limited. Integrating ethnomedicinal knowledge with systematic research could guide future cancer therapies and inform policy development in integrative oncology.

## Introduction

Cancer remains a leading cause of morbidity and mortality worldwide, accounting for nearly one in six deaths globally. Despite significant advances in diagnostics and therapeutics, conventional cancer treatments such as chemotherapy, radiation, and surgery are often associated with adverse effects, resistance, and high economic burden. These limitations have prompted growing interest in complementary and integrative approaches, including the use of plant-based therapies rooted in traditional medicine systems (1).

India, with its rich biodiversity and longstanding traditions of ethnomedicine, offers a vast pharmacopeia of medicinal plants with therapeutic potential (2). In particular, the southern state of Tamil Nadu harbors a deep legacy of herbal healing practices, sustained by intergenerational knowledge among Yoga & Naturopathy, Siddha physicians, local vaidyas, and indigenous communities. While many of these herbs have been used for centuries to manage a variety of ailments including tumors and growths their scientific validation in the context of cancer remains incomplete.

This narrative review aims to bridge the gap between traditional herbal knowledge and modern scientific inquiry by examining 32 herbs widely used or recommended for their purported anticancer effects by a collective of herbal activists, including physicians and traditional healers from Tamil Nadu. The primary objective is to evaluate the scientific evidence regarding their efficacy in cancer prevention and treatment, both as standalone interventions and as adjuncts to standard therapies. The secondary objective is to elucidate the underlying mechanisms of action, focusing on phytochemical constituents and their interactions with cancer cell biology.

Indigenous people in India have long used medicinal plants to fight cancer. However, most such herbal treatments remain anecdotal and under-researched. Tavakoli et al. reported that many herbal cancer therapies have not undergone systematic scientific evaluation (3). By beginning with community‐endorsed remedies rather than familiar botanicals, this review inverts the usual research paradigm and systematically examines what evidence exists for each plant. This practice to literature approach sets this review apart, highlighting unique leads from Tamil Nadu’s tradition while exposing critical gaps in the scientific validation of these anticancer claims. In doing so, it emphasizes the critical need to integrate ethnobotanical knowledge with rigorous biomedical research, thereby contributing to a more inclusive and evidence-informed framework for integrative oncology.

## Methods

### Study design

This narrative review employed a comprehensive approach to examine 32 herbs with purported anticancer properties, identified by a group of herbal activists, including physicians and traditional healers from Tamil Nadu, India. The term “herbal activists” refers to a collaborative group comprising physicians trained in traditional medicine, local healers, and community practitioners actively engaged in preserving and promoting indigenous herbal knowledge. Their collective input was used as a pragmatic starting point to identify frequently utilized herbs warranting scientific validation. The selection of these herbs was based on anecdotal evidence, popular use, and their anthropological prevalence. The primary objective was to evaluate existing literature on the efficacy of these herbs, used either independently or alongside standard cancer treatments. The secondary objective was to explore the mechanisms by which their phytochemicals influence cancer metabolism.

### Database and search strategy

A literature review was conducted across PubMed, Scopus, and Google Scholar, covering the period from 2014 to January 2025. Manual screening of references from key studies complemented the database search. The search aimed to identify at least five published articles per herb, irrespective of publication type. Keywords combined scientific and common names of herbs with terms such as “anticancer properties,” “cytotoxicity,” “traditional medicine,” “phytochemicals,” “tumor inhibition,” “herbal oncology,” “integrative cancer therapies,” and “bioactive compounds.” Boolean operators were applied to optimize search precision: AND linked different concepts (e.g., “herb name AND anticancer activity AND cytotoxicity”), OR connected synonyms (e.g., “phytochemicals OR bioactive compounds”), and NOT excluded irrelevant results (e.g., “traditional medicine NOT Ayurveda”).

### Eligibility criteria

**Figure 1:**
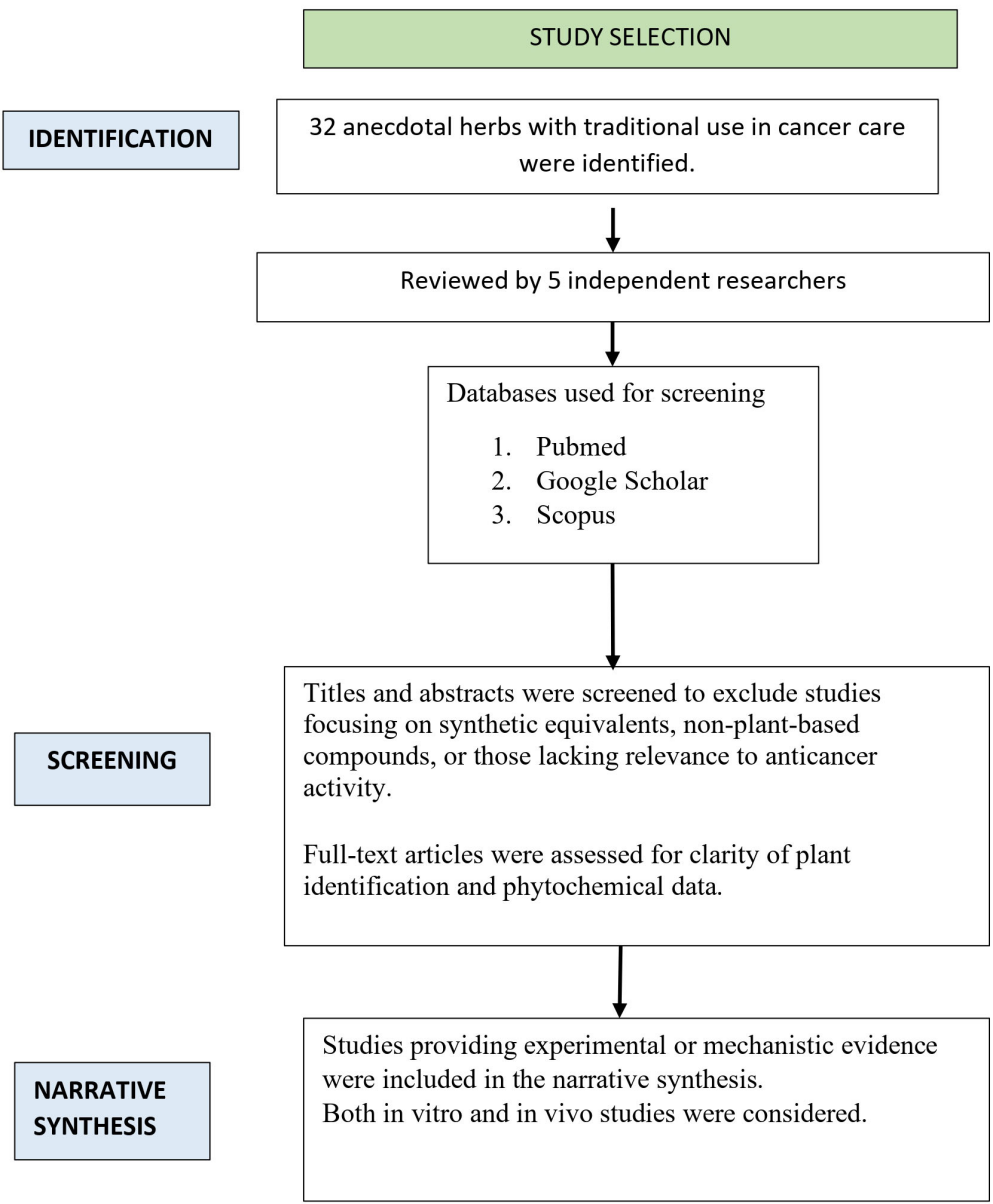
Overview of narrative synthesis process.

Studies published between 2014 and January 2025, available in full text and written in English, were included. Eligible studies specifically investigated the anticancer effects of the 32 identified herbal plants or their bioactive constituents. Both preclinical (*in vitro*, *in vivo*, and in silico) and clinical studies were considered. Exclusion criteria included studies unrelated to cancer, duplicate publications, and literature reviews. **Figure 1** illustrates the flow of the literature review used for this narrative review.

### Data extraction

Five independent reviewers conducted a two-stage evaluation of selected studies, beginning with title and abstract screening, followed by full-text review. Discrepancies were resolved through discussion or consultation with an additional reviewer. Extracted data included study characteristics such as the investigated herbs, cancer types and corresponding cell lines, and strength of evidence (preclinical or clinical). Additional details encompassed the botanical and local (Hindi) names of herbs, plant parts used, mode of administration, major bioactive phytochemicals with reported anticancer activity, mechanisms of action (e.g., apoptosis induction, inhibition of angiogenesis and metastasis), cancer models employed (*in vitro*, *in vivo*, or in silico), and main study outcomes.

### Data charting process

A standardized data-charting template was developed collaboratively by five reviewers using Microsoft Excel to ensure systematic extraction. Each reviewer independently entered and verified study data, and the template was refined through iterative rounds of review and consensus to enhance accuracy, completeness, and consistency across all variables.

## Results

**Table 1** summarize the general overview of the herbs discussed in this review. This review analyzed preclinical, *in vivo*, and clinical studies related to 32 traditionally used anecdotal herbs to determine whether their applications are supported by evidence-based research. **Supplementary Table 1** summarizes the available preclinical and clinical evidence regarding the anticancer potential of these herbs. The independent effects of each plant on cancer biology are detailed below.

**Table 1:** Ethnobotanical overview of selected Indian Herbs used for cancer.

Botanical name	Local name (Hindi)	Parts used	Mode of use
Annona muricata	Hanuman phal (हनुमान फल)	Seed, leaves, barks, stems, roots and flowers	Leaf decoction, ethanolic extract, seed oil
Simarouba glauca	Laxmitaru (लक्ष्मीतरु)	Bark, Leaves (twigs)	Methanolic leaf extract, leaf decoction, Powder
Catharanthus roseus	Sadabahar (सदाबहार)	Leaf, Stem, Seed, root and Petal	Alkaloid extract (vincristine, vinblastine)
Azadirachta indica	Neem (नीम)	Seeds, leaves, flowers, and fruits	Leaf/seed extracts, Neem oil
Citrus limon	Nimbu (नींबू)	Fruit (peel, juice)	Peel oil (D-limonene), Fresh Juice
Justicia gendarussa	Nili Nirgunthi (निली निर्गुण्ठी)	Leaves and roots	Methanolic leaf extract, Decoction
Murraya paniculata	Kamini (कामिनी)	Leaves, shoots, twigs, root, flower	Juice/Extract
Murraya koenigii	Kari patta (करी पत्ता)	Leaves	Mahanine-enriched extract, leaf powder (capsules, decoction)
Hibiscus sabdariffa	Gudhal (गुड़हल)	Calyces (flowers)	Aqueous extract, herbal tea, juice
Centella asiatica	Mandookaparni (मण्डूकपर्णी)	Leaves (whole plant)	Fresh juice, extract (asiatic acid)
Morinda citrifolia	Noni (नॉनी)	Fruit (berries)	Juice, extract, capsules
Pimenta dioica	Allspice (ऑलस्पाइस)	Berries (dried)	Berry essential oil (eugenol), ethanol extract
Cynodon dactylon	Durva (दूर्वा)	Root (whole grass)	Decoction, methanolic extract, fresh juice
Zizyphus nummularia	Jharberi (झरबेरी)	Leaves, fruits, seeds, bark, roots, flower	Decoctions or powders
Curcuma longa	Haldi (हल्दी)	Rhizome	Rhizome powder (oral) or paste, decoction, ointment
Aloe barbadensis	Ghritkumari (घृतकुमारी)	Leaf (Fillet, Mucilage, and Rind) and Flower	Oral (juice) or topical (gel/ointment) of leaf juice/gel
Gymnema sylvestre	Gurmar (गुड़मार)	Roots, stem, and leaves	Leaf extracts or teas (juice/powder)
Gloriosa superba	Kalihari (कलिहारी)	Tubers	Alkaloid extracts from tubers
Piper nigrum	Kalimirch (काली मिर्च)	Fruit (peppercorn)	Dried fruit/spice; extracts
Coriandrum sativum	Dhaniya (धनिया)	Leaves, root and seeds	Leaves (herb) and seed (spice); decoctions
Phyllanthus emblica	Amla (आंवला)	Fruit and seeds	Fruit juice/extract; decoction
Cucurbita pepo	Safed kaddu (सफ़ेद कद्दू)	Fruit seeds and Flowers	Juice, Seed oil; extracts
Prunus dulcis	Badam (बादाम)	Seed (almond)	Nuts (raw/oil)
Cyamopsis tetragonoloba	Gwar (ग्वार)	Seeds (cluster bean)	Dried flour, guar gum (from seeds)
Anisomeles malabarica	Van Tulsi (वन तुलसी)	Leaves (herb)	Whole-plant extracts
Tridax procumbens	Ghamra (घमरा)	Leaves, flowers	Leaf extracts
Cuminum cyminum	Jeera (जीरा)	Seeds (cumin)	Spice (seed); decoction
Trigonella foenum-graecum	Methi (मेथी)	Leaves, seeds	Seeds (spice), leaf extract
Solanum nigrum	Makoi (मकोई)	Leaves, berries, stem	Extracts (decoction)
Cucumis sativus	Kheera (खीरा)	Fruit, seeds	Fruit (raw, juice)
Piper betel	Paan (पान)	Leaves	Chewed raw (betel quid) or extracts
Withania somnifera	Ashwagandha (अश्वगंधा)	Root, leaves, stems	Decoction, powder of dried root, capsules/tablets

### Annona muricata

*Annona muricata* (soursop) has shown notable anticancer effects in preclinical studies, primarily attributed to phytochemicals like annonaceous acetogenins and alkaloids (4). Leaf extracts, along with seed and bark of soursop in some studies, have demonstrated activity against cancer cell lines such as MCF-7 (breast), HT-29 (colorectal), PC-3 (prostate), and PANC-1 (pancreatic) (5–11). The main mechanism involves inhibition of mitochondrial Complex I, leading to ATP depletion and selective cancer cell death; apoptosis is triggered via upregulation of Bax, caspase-3, and caspase-9, and downregulation of Bcl-2 (6, 12–15). The extracts of soursop leaves also induce cell cycle arrest at G0/G1 or G2/M phases, likely through cyclin-CDK regulation (16–19), and promote oxidative stress via ROS generation, causing DNA damage (7, 20–22). Furthermore, inhibition of NF-κB, PI3K/Akt, and EGFR pathways reduces proliferation, invasion, and angiogenesis (23–25).

### Simarouba glauca

*Simarouba glauca* (paradise tree) has demonstrated broad-spectrum anticancer activity, primarily in *in vitro* models. Extracts from leaves, bark, and seeds, prepared using methanol, petroleum ether, or chloroform contain phytochemicals such as alkaloids, flavonoids, terpenoids, glycosides, triterpenoids, and quassinoids, exhibiting cytotoxic, antiproliferative, and pro-apoptotic effects. Leaf extracts of S. glauca showed potent cytotoxicity in leukemia and non-small cell lung cancer cells via caspase-dependent apoptosis, activating both mitochondrial and death receptor pathways (26, 27). In breast cancer models, ethanolic and methanolic extracts of S. glauca displayed low half-maximal inhibitory concentration (IC_50_) values and similar apoptotic mechanisms (28).Bark extracts of S. glauca disrupted oxidative phosphorylation and induced apoptosis in triple-negative breast cancer cells (29), while chloroform and ethyl acetate fractions suppressed cell proliferation in cervical, colorectal, and mammary cancer cell lines via oxidative stress and apoptotic pathway activation (30). *In vivo*, Yeo et al., reported synergism between gemcitabine and glaucarubinone from *S. glauca* seeds, suppressing pancreatic tumor growth through HIF-1α and β-catenin regulation (31). Additional studies in bladder, prostate, and colorectal cancers revealed mechanisms such as p53/p21 upregulation, G0/G1 arrest, and mitochondrial depolarization (32–34), affirming *S. glauca*’s anticancer potential.

### Catharanthus roseus

*Catharanthus roseus* is a well-documented medicinal plant known for its anticancer alkaloids vincristine and vinblastine, widely used in chemotherapy for leukemia, Hodgkin’s lymphoma, and breast cancer. These compounds act by inhibiting mitosis, disrupting microtubule formation, and inducing apoptosis (35, 36). An *in vitro* study reported the cytotoxicity effects of methanolic leaf extracts of *C. roseus* against MCF-7 breast cancer cells indicating potential anti-carcinogenic effect of C. roseus (37). Endophytic fungi such as *Talaromyces radicus* and *Eutypella* spp., isolated from *Catharanthus roseus*, have been shown to produce vincristine and vinblastine like compounds. These fungi induce reactive oxygen species (ROS) generation and disrupt mitochondrial membrane potential, thereby mimicking the apoptotic effects of the parent alkaloids (38, 39). A 2023 *in vivo*–*in vitro*–in silico study using a nanoemulsion formulation of incensole acetate from *C. roseus* essential oil demonstrated significant anticancer activity against breast cancer, with improved bioavailability and reduced toxicity (40). Similarly, an *in vitro* study on lung adenocarcinoma cells confirmed *C. roseus* induced cytotoxic and apoptotic effects (41). Furthermore, an *in vivo* study combining *C. roseus* with *Phyllanthus niruri* showed enhanced macrophage polarization and immune response in mice with induced breast cancer (42).

### Azadirachta indica

*Azadirachta indica* (neem) is a medicinal plant rich in over 300 bioactive compounds, including azadirachtin, gedunin, nimbin, nimbolide, and quercetin, with established antineoplastic properties. Extracts from various parts of the plant exhibit cytotoxic, anti-proliferative, and pro-apoptotic effects across multiple cancers (43). Methanolic bark extracts of neem modulate migration-related genes (*ZO-1*, *MMP2*, *FAK*, *N-cadherin*) in cervical cancer (44), while specific compounds such as phthalic acid and 4-ethylbenzamide show cytotoxicity in breast cancer (45). Nimbolide, another bioactive compound in neem has shown to inhibit PI3K/Akt/mTOR and ERK pathways in pancreatic cancer, suppresses epithelial-mesenchymal transition, and induces mitochondrial-mediated apoptosis (46). Other compounds like desacetyl nimbinene and epoxyazadiradione modulate MAPK and PI3K/Akt signaling, leading to apoptosis and reduced tumor growth (47). Gedunin targets the Hedgehog pathway in pancreatic cancer and promotes apoptosis via p53 and Bax in teratocarcinoma (48, 49). Neem-derived flavonoids and limonoids also inhibit STAT3, BCL-2, and enhance *BAX*, *Caspase-3*, and mitochondrial depolarization in hepatocellular and prostate cancers (50, 51). Additionally, neem silver nanoparticles downregulate *VEGF* and *Cyclin D1* in lung cancer (52), and combined extracts show apoptotic effects in breast, prostate, and colorectal cancers through p53/Bax activation and BCL2 inhibition (50).

### Citrus limonum

*Citrus limonum* (lemon) contains diverse bioactive compounds including flavonoids (naringenin, naringin, quercetin, nobiletin, 5-demethylnobiletin, 2′-hydroxyflavanone, tangeretin), alkaloids (synephrine, N-methyltyramine, hordenine), terpenoids (limonene, geranial, neral, obacunone), and secondary metabolites like tannins, saponins, and modified citrus pectin (MCP). MCP inhibits STAT3 phosphorylation and galectin-3, suppressing M2 macrophages and STAT3 signaling in breast, prostate, and ovarian cancers (53). Lemon juice–derived nanovesicles show antileukemic effects via TRAIL and anti-angiogenesis mechanisms (54). Flavonoids modulate estrogen signaling, inhibit aromatase, reduce BCL-2, and activate caspases in breast cancer (55). Nobiletin and its derivatives regulate G0/G1 arrest, apoptosis, and multiple signaling pathways across cholangiocarcinoma, colorectal, lung, gastric, and myeloid cancers (56). Other compounds like 2′-hydroxyflavanone, tangeretin, and obacunone exhibit anti-STAT3, anti-inflammatory, and antiproliferative effects (57), while alkaloids and terpenoids contribute to cytotoxicity in hepatocellular, cervical, and melanoma models via redox modulation (58).

### Justica gendarussa

*Justica gendarussa* is a member of Acanthaceae family, which contains bioactive compounds such as flavonoids (including kaempferol and naringenin), alkaloids, triterpenoidal saponins, amino acids, aromatic amines, stigmasterol and lupeol. Kaempferol and naringenin have shown cytotoxic effects against mammary carcinoma (59), while methanolic leaf extract exhibits both proapoptotic and cytotoxic activity in lung cancer (60). Gendarusin A is likely to activate multiple cell signaling pathways related to programmed cell death, including the expression of proteins such as BID, BAX and BCL-2 in T-lymphocyte cancer (61).

### Murraya paniculata

*Murraya paniculata* (Orange Jessamine) contains a wide range of bioactive compounds including flavonoids, phenols, alkaloids, coumarins, polysaccharides, and essential oils rich in sesquiterpenes like β-caryophyllene, limonene, and spathulenol (62). Flavonoids and coumarins inhibit metastasis by suppressing cancer cell adhesion and invasion (63). A specific flavonoid glycoside modulates integrin β1, EGFR, COX-2, MMPs, EMT markers, and STAT3/NF-κB/PI3K pathways in lung cancer (64). Compounds such as 6′-O-β-d-apiofuranosylapterin and coumarins showed cytotoxicity against leukemia, lung, liver, breast, and colon cancer cells (65). Sesquiterpenes showed hepatotoxicity in liver cancer models (62), while certain coumarins (e.g., murpanidin, murralongin) inhibited colon cancer metastasis by downregulating EpCAM without cytotoxicity (66). Alkaloids, saponins, phenols, and volatile oils induced apoptosis and inhibited growth in breast cancer (67). Coumarin derivatives like auraptene and scopolin exhibited antiproliferative activity via estrogen receptor binding (68). Nanoparticle formulations enhanced cytotoxicity by targeting DNA replication in MCF-7 cells (69).

### Murraya koenigii

*Murraya koenigii* (curry leaf plant) contains potent bioactive compounds, especially carbazole alkaloids such as mahanine, mahanimbine, and koenimbine, which exhibit cytotoxic, anti-proliferative, apoptotic, anti-inflammatory, and anti-metastatic properties across various cancer types. Mahanine induces apoptosis via mitochondrial pathways, increases ROS, activates caspases 3, 7, and 9, upregulates Bax, downregulates Bcl-2, and targets estrogen receptor alpha and CDKs in leukemia and breast cancer cells (70). It also interferes with STAT3, PI3K/AKT, and Wnt/β-catenin signaling, contributing to cell cycle arrest and tumor suppression in lung, ovarian, and prostate cancers (71–73). Grinimbine and other alkaloids such as koenimbine and mukonal induce G0/G1 arrest and apoptosis, demonstrating selective cytotoxicity in colorectal (HT-29), breast (MCF-7, MDA-MB-231), and glioblastoma cells (U373MG) (74).These compounds also reduce cell viability, disrupt mitochondrial membrane potential, deplete glutathione, modulate heat shock proteins (HSP70/90), and increase DNA damage in hepatocellular carcinoma (HepG2) (75).

### Hibiscus sabdariffa

*Hibiscus sabdariffa*, a member of the Malvaceae family, is rich in diverse bioactive compounds including gossypetin, oleuropein, hydroxytyrosol, flavonoids, anthocyanins (such as cyanidin and delphinidin), catechin, ellagic acid, protocatechuic acid, quercetin, hibiscus acid, rutin, oleanolic acid derivatives, and polysaccharides. These compounds exert anticancer effects across various cancer types through multiple mechanisms. Gossypetin induces cell cycle arrest in oral cancer cells by upregulating p21 (76), while oleuropein forms copper complexes that trigger apoptosis in neuroblastoma cells (77). Polyphenol-rich extracts downregulate ERα and BRCA1, promote autophagy, and inhibit proteasome activity in breast cancer cells (78). Fatty acid esters selectively induce apoptosis in colon cancer by targeting the 2HQ6 protein (79), and hibiscus acid along with anthocyanins inhibits the proteasome in multiple myeloma cells (80, 81). Catechins and flavonoids suppress prostate cancer progression by inhibiting Akt/NF-κB/MMP-9 signaling (82). In breast and oral cancers, anthocyanins and rutin reduce oxidative stress, protect against DNA damage, and induce cytotoxicity (83, 84).

### Centella asiatica

*Centella asiatica* (mandukparni/gotu kola) contains triterpenoids (asiaticoside, madecassoside, asiatic acid) and flavonoids (quercetin, kaempferol) that exhibit anticancer, anti-angiogenic, cytotoxic, and apoptotic effects across multiple cancers. These compounds inhibit VEGF165-induced angiogenesis and modulate key signaling pathways like PI3K/Akt, PD-1, RAGE, and AGE-1. In oral, liver, and melanoma models, they induce apoptosis via Bax/Bcl-2 regulation, caspase activation, and suppression of cAMP-PKA-MITF and migration pathways (85). Asiatic acid enhances mitochondrial damage, modulates YAP1, VEGFA, STAT3, and inhibits EMT (86, 87), while also suppressing PI3K/Akt/mTOR signaling to promote autophagy and apoptosis in lung, colon, and ovarian cancers [88–94].Phytochemicals such as alkaloids, glycosides, and flavonoids induce mitochondrial dysfunction, oxidative stress, and caspase-mediated apoptosis in MCF-7, A549, HeLa, and Caco-2 cells (95).

### Morinda citrifolia

*Morinda citrifolia* (noni), from the Rubiaceae family, is rich in bioactive compounds such as scopoletin, epicatechin, phytosterols, nordamnacanthal, and damnacanthal, contributing to its antioxidative, immunostimulatory, hepatoprotective, anti-inflammatory, and anticancer activities. Epicatechin and scopoletin induce apoptosis in leukemia (Jurkat, WEHI-3b) and lung adenocarcinoma (A549) through caspase-3/8 activation, G0/G1 arrest, and downregulation of tumor proliferation genes (EGFR, MDM2, RAF1, mTOR), while enhancing immune responses and reducing COX-2 expression (96). Nordamnacanthal and damnacanthal exhibit apoptotic and anti-proliferative effects in breast cancer (MCF-7, MDA-MB231, 4T1), promoting annexin V+ expression, G1 arrest, and modulation of apoptotic regulators such as p53, Bax, Bcl-2, XIAP, and ER-α (97–99). Other phytoconstituents like asperuloside, asperulosidic acid, deacetylasperulosidic acid, eugenol, rutin, and morindone target MAPK6 and MDM2 pathways, suppressing tumor growth and promoting p53-mediated apoptosis in colorectal and liver cancers (100, 101).Collectively, Morinda citrifolia exerts anti-cancer effects via modulation of multiple apoptotic and inflammatory pathways including VEGF/EGFR/NF-κB, AKT1, MAPK, JAK2/STAT3/STAT5A, contributing to reduced tumor growth and angiogenesis (102).

### Pimenta dioica

Commonly known as allspice, the aqueous extract of *Pimenta dioica* has been identified as an anti-cancer agent against prostate cancer and identified a potent novel anti-proliferative compound Ericifolin (eugenol 5-O-beta-galloylglucopyranoside. Ericifolin, gallic acid and eugenol derivatives have demonstrated anti-tumor, apoptotic, cytotoxic, chempreventive and anti-proliferative properties by increasing the levels of autophagy markers LC3B and LC3B-positive puncta and downregulating Akt and mTOR phosphorylation in breast cancer (triple negative breast cancer, TNBC – MDA-MB231 cells) (103).

### Cynodon dactylon

*Cynodon dactylon*, commonly known as Bermuda grass is valued in Indian traditional medicine for treating cancer, with studies identifying 22 compounds including hydroquinone, levoglucosenone and furfural as major constituents responsible for its medicinal properties. The methanolic extract from root exhibited anti-tumor, anti-inflammatory and chemo preventive effects by modulating the levels of liver detoxification enzymes and by decreasing the levels of serum glutamate pyruvate transaminase and serum glutamate oxaloacetate transaminase in Liver cancer (Hepatocellular carcinoma induced by diethyl nitrosamine) (104). The petroleum ether extract from leaf, stem and root exhibited cytotoxic, apoptotic, antitumor and antiproliferative effects confirmed via DNA fragmentation assay in Laryngeal cancer (Hep-2) Cervical cancer (HeLa) and Breast cancer (MCF-7) (105). Hydroxycinnamic acid, alkaloids, flavonoids, phenolic compounds, tannins, steroids and vitamin A extracted from the leaves exhibited cytotoxicity through apoptosis, antiproliferative, antitumor and antioxidant activity likely contributing to chemo preventive effects in chronic lymphocytic leukemia (K-562 cells line) (106).

### Zizyphus nummularia

Commonly known as Indian Jujube, exhibits significant anti-cancer activity against multiple cell lines including human colon adenocarcinoma (HT-29), breast cancer (MCF-7), ovarian cancer (OVCAR-3), leukemia (K-562), human kidney carcinoma (A-498) and pancreatic Capan-2 cancer cells. Its ethanolic extract contains compounds like Lupeol, Rutin, Caryophyllene, Procyanidin B1, Nummularine R, 2-Isobutyl-3-methoxypyrazine, Nummularine A, Luteolin-7-O-glucoside. The ethyl Acetate Extract includes Guaifenesin, Nummularine R, Quercetin, Procyanidin B1, 2-Methoxy-4-vinylphenol, Coumaroylquinic acid, Chlorogenic acid. These constituents demonstrate anti-tumor, apoptotic, cytotoxic and chemopreventive effects by destabilizing the microtubule network in cancer cells (KAIMRC2) and upregulated phosphorylated mTOR and AKT proteins which indicates a possible involvement in signaling pathways related to cell growth and survival (107). Quercetin and kaempferol further enhance ROS generation, leading to apoptosis, while upregulating p38 MAPK, p21 and p27 and downregulating MMP-9 to inhibit metastasis (108). The ethanolic extract also suppresses ERK1/2 (MAPK) and NF-KB signaling pathways, downregulating alpha 2 integrin expression, decreasing VEGF production, reducing nitric oxide levels and upregulating E-cadherin which is a tumor suppressor protein (109).

### Curcuma longa

Turmeric (*Curcuma longa*), extensively mentioned in Ayurvedic literature, demonstrates wide-ranging therapeutic effects due to bioactive constituents like curcumin, demethoxycurcumin, bisdemethoxycurcumin, tumerone, ar-tumerone, and quercetin (110). Curcumin modulates key oncogenic pathways such as RAS-ERK, PI3K/AKT/mTOR, COX-2, NF-κB, and JAK/STAT, showing efficacy in neurofibromatosis type 1, breast, cervical, lung, esophageal, uterine, prostate, pancreatic, hepatocellular, and colorectal cancers (111). Tumerone enhances curcumin bioavailability and ar-tumerone induces apoptosis in cervical cancer cell lines (110). Curcumin also potentiates the effects of bortezomib, paclitaxel, and FOLFOX chemotherapy, and suppresses tumor-associated inflammation and angiogenesis (112). Clinical studies report tolerability and efficacy of curcumin-based interventions in breast, prostate, and colorectal cancers, including oral doses up to 6000 mg/day and topical application for radiation-induced oral mucositis (113). Quercetin, an additional bioactive, shows promise for further exploration due to its high binding affinity in silico and anti-cancer potential (114). Collectively, turmeric exerts its anti-cancer properties by disrupting cell proliferation, inducing apoptosis, modulating immune responses, and targeting angiogenesis, EMT, and cancer stem cell pathways.

### Aloe barbadensis

*A. barbadensis* Miller (Aloe vera), commonly studied species of Aloe (115), is known for many properties like cytotoxicity (115–118), anticancer (115, 117–120), antitumor (115, 118), genotoxic (118), antiproliferative and apoptotic (120). Aloctin, a lectin from *Aloe vera* (AV), has been reported to influence autophagy and apoptosis in various human cancer cell lines, including AGS (gastric adenocarcinoma), HCT116 (colon cancer), HEP3B (hepatoma), HL60 (acute promyelocytic leukemia), K562 (chronic myelogenous leukemia), and Saos-2 (osteosarcoma) (115). A synergistic effect of *A. vera* and *A. arborescens* displayed antiproliferative activity against HT-29 cells via MMP-2 and MMP-9, underlining the need to explore other species (119). Aloin downregulated cyclin B1 in MCF-7 cells and inhibited topo IIα expression in both MCF-7 and SKBR-3 cell lines (116). A preclinical study showed that *A. vera* gel exhibited stronger anticancer effects against melanoma cells than its purified form, suggesting greater potency of the natural extract and supporting its use as a protective and skin-healing agent (117). Lyophilized *A. vera* extract induced cytotoxicity in HepG2 cells by upregulating p53 and downregulating Bcl-2 (118). In A549 and HT-29 cells, combining royal jelly with *A. vera* enhanced efficacy and reduced AV toxicity (120).

### Gymnema sylvestre

*Gymnema Sylvestre* (GS) or cowplant exhibits diverse properties including hepatoprotection (121), antioxidant (121–123), anticancer (124–130), immunomodulation (121, 125), apoptotic (122, 129, 130), autophagy (128), antiproliferation (126, 130), cytostatic (128), chemoprevention (123), cytotoxic (50, 122, 124, 126), and genotoxicity (124). These effects are mediated by multiple components like alkaloids, steroids, flavonoids (127), saponins (122, 127), triterpines (121, 127), gymnemagenol, dasyscyphin C (126) and gymnenic acid (125). An *in vivo* study demonstrated that gavage with GS triterpenoid saponin extract (GST) could upregulate IL-2 and IL-4 mRNA expression and reduce TNF-α expression indicating immunomodulation in breast cancer cell lines- MCF-7, MDA-MB-231 (121) along with tumor weight reduction at dose 200mg/kg body weight per day in MCF-7 and MDA-MB-468 cell lines (122). The ethanolic extract displayed apoptotic activity against A375 cells by activating mitochondria- dependent cell death pathway shown by increase in cytochrome c, caspase 3, PARP, Bax and reduced ICAD, EGFR and Bcl2 (130). Silver nanoparticles of GS demonstrated inhibition of cell growth in HT29 human colon adenocarcinoma cells which could have been by intracellular reactive oxygen species generation (127) or by cell cycle arrest (129). GS extract also displayed considerable cytotoxic effect and autophagy in Human continuous Glioblastoma cell line U87Mg (128) and chemoprevention in papillomas (123). It presented a significant synergy with neem and Moringa Oliefera on A549 lung cancer cells (50).An active principle of GS, gymnemagenol highly inhibited growth of HepG2 cells, while some compounds inhibited by inducing apoptosis (124, 126). GS leaf extract, via gymnemic acid, enhances macrophage activity and supports myeloid and lymphoid immune components, aiding in restoring innate immune function (125).

### Gloriosa superba

*Gloriosa superba*, or kanivalu kilangu, contains phytochemicals like colchicine, gloriosine, and thiocolcoside that display various bioactivities, including anticancer, apoptotic, and anti-inflammatory effects. Colchicine exhibits strong anticancer properties in lung, breast, and liver cancers *in vitro*, and promotes IL-8 activity in pancreatic cancer (131). Gloriosine inhibits migration and induces apoptosis in cancer cells, such as A549 lung cancer cells (132). Synthetic derivatives selectively inhibit tumor cells and induce apoptosis, while suppressing NF-κB activity. Purified colchicine is minimally cytotoxic at low doses but shows resistance at higher concentrations (133). It affects MDA-MB231 cells at 40 nM, and other cancers at 80 nM (134). G. superba’s rhizome enhances anticolon activity through p53 upregulation and NF-κB downregulation (135). Phytogenic platinum and palladium nanoparticles from G. superba tuber extracts show significant anticancer effects, especially in MCF-7 breast cancer cells, causing apoptosis and promoting free radical formation (136). Thiocolchicoside from the seeds inhibits osteoclastogenesis by blocking NF-κB activation, offering potential in treating metastatic bone disease (137). The plant’s methanolic extracts have strong antioxidant properties and effectively inhibit Hep-G2 liver cancer cells. Colchicine disrupts the mitotic spindle apparatus, impacting cells with high metabolic rates (88). Thereby, Gloriosa superba shows anticancer potential by promoting apoptosis, reducing NF-κB activity, and inducing oxidative stress in cancer cells.

### Piper nigrum

*Piper nigrum* or commonly known as pepper or “King of Spices” elicits beneficial effects on various conditions due to the presence of active principles like piperine, Piperettine, Trichostachine, Piperine, Piperolein A, Piperolein B (89). Pepper displayed apoptotic, anticancer, antiproliferative activity through JNK/p38 MAPK-mediated intrinsic apoptotic pathway in A2780 cells- human ovarian cancer cells (90). Cytotoxicity enhanced when combined with turmeric on lung cancer cell lines (91), while in metastatic 4T1 breast cancer and B6-F10 melanoma, PN enhanced antitumor responses by promoting CD45^+^ hematopoietic cell infiltration and modulating the Th1/Th2/Treg ratio (92). A further aspect worth noting is that PN extract revealed apoptotic pathways in colorectal cancer cell line, which produced antitumor activity by mitigating Matrix metalloproteinases(MMP). The mode of administration notably intragastric with doses- 25, 50, 100mg/kg/day paves a way for natural anti-colorectal cancer drug formulations (93). A preclinical study depicted anticancer activity by suppressing TYR and TRP-1 genes in melanoma cells (94). Piperine also helps enhance the effect of anticancer drugs by suppressing level of ABCB1, ABCC1 and ABCG2 genes which encode P-gp, MRP1 and BCRP respectively in line MCF-7 cell line and its doxorubicin resistant subline MCF-7/DOX and A-549 cell line and its resistant subline A-549/DDP which suggests that Piperine may reverse multi-drug resistance (138). Further, Piperine inhibited H. pylori growth and motility and adhesion to gastric adenocarcinoma by suppressing Flha and flge expression (139).

### Coriandrum sativum

*Coriander sativum* also known as coriander is an aromatic, edible plant, commonly used as a spice and used chiefly in traditional medicines (140, 141). It has an umbrella of active principles including polyphenols like catechin, epicatechin, epicatechin gallate, vanillic acid, components like adenine, adenosine, tryptophan, coriandrin, alkaloids, flavonoids, tannins, saponins and alpha linalool among many others (141–144). Coriander seeds oil and nanoemulgel along with doxorubicin were reported to effectively inhibit cancer cell lines like MCF-7, Hep3B and HeLa, thus exhibiting its cytotoxicty (140). Antileukemic activity was explicitly seen as synergestic action of catechin and rutein components in a preclinical study with *in vivo* dosage of 2000mg/kg per oral (141). A intriguing study with various concentrations of 1%, 3%, and 5% w/w dietary coriander powder showed significant anticancer effects in HepG2 and B16F10 cells by reducing migration, invasion, and inhibiting MMP-2 and u-PA activities (145). CS induced apoptosis in SH-SY5Y neuroblastoma cells via the mitochondrial apoptotic pathway by increasing Bax and reducing Bcl-2 expression (142). In human colon cancer HT-29 cell line, the extract reduced the viability of cancer cells in concentration dependent manner. 267 Alpha-linalool from decoction of coriander root displayed reduction in β-catenin and TGF-β/SMAD pathway genes like P-GSK-3β, TGF-β, and P-SMAD2/3 and reduction in tumor growth (144).

### Phyllanthus emblica

*Phyllanthus emblica* L. (Indian gooseberry or amla) is a nutrient‐dense functional fruit whose vitamin C, polyphenols (e.g. gallic/ellagic acids, flavonoids, tannins) and other phytoconstituents exert broad anticancer effects. Bioactive compounds such as Trigonelline, Naringin, Kaempferol, Catechin, Quercetin, Embinin, Isorhamnetin, Apigenindin and Colchicine downregulated c-Myc and cyclin D1, suppressed beta-catenin signaling and caused p53-independent apoptosis (increased Bax/Bcl-2) in colon cancer (colon cancer stem cells, HCT116) (146). Likewise, compounds like Gallic acid, syringic acid, ellagic acid, catechin, epicatechin, trans-cinnamic acid, rutin, condensed and hydrolysable tannins, polyphenols and flavonoids inhibited preneoplastic lesions, enhanced antioxidant enzymes (catalase, GPx), reduced oxidative stress, downregulated PCNA+ cell proliferation and modulated xenobiotic metabolism in liver and colon (dual carcinogenesis model) (147). Gallic acid, corilagin and ellagic acid induced apoptosis, inhibited tumor volume and weight, sustained drug release and enhanced bioavailability via SLNs in lung cancer (Lewis lung carcinoma model) (148). Extracts from the fruit increased ROS production, enhanced mitochondrial membrane potential, upregulated apoptotic genes (BAX, CASP3), modulated antioxidant (SOD2, GPX3) and inflammatory genes (IL6, IL-1 beta, TNF-alpha, TGF-beta) in chronic lymphoblastic leukemia (CLL) (149). Collectively, these findings indicate that P. emblica’s bioactives inhibit tumor progression and recurrence across multiple cancers by scavenging ROS, modulating redox and inflammatory pathways, and reprogramming oncogenic and epigenetic signals to induce apoptosis and block proliferation.

### Cucurbita pepo

*Curcubita pepo*, commonly known as Pumpkin is native to northern Mexico and USA. Bioactive compounds such as flavonoids, triterpenoids, steroids, tannins, phytosterols, and saponins have been shown to modulate oxidative stress markers (↑SOD, CAT, GSH; ↓MDA, nitrite), reduce aberrant crypt foci (ACF) counts, and histological restoration in colon cancer thereby demonstrating anti-tumor, chemopreventive and antioxidant properties (150). Ribosome-inactivating proteins (cucurmosins) and cucurbitacins from pumpkins induce cell-cycle arrest (G0/G1 or G2/M) and caspase-dependent apoptosis in various tumor cells (e.g. HER2+ breast, NSCLC, colorectal), while inhibiting oncogenic pathways (Notch–Hes1, PI3K/Akt/mTOR, STAT3) and activating AMPK (151, 152). Likewise, major fatty acids from pumpkin; Oleic acid, Stigmasta-7,25-dien-3-ol and Linoleic acid mediated apoptosis via chromatin condensation, membrane blebbing, nuclear fragmentation and modulates antioxidants stress in papillary thyroid carcinoma (153).Pumpkin carotenoids such as Beta-carotene, alpha-carotene, lutein, zeaxanthin, violaxanthin, antheraxanthin and esterified carotenoids induced cell death at high concentration possibly mediated by ROS cytotoxicity in neuroblastoma (SH-SYS cells) (154). Emerging formulations also show promise: green-synthesized Cu–Mn nanoparticles (using pumpkin seed extract) induced DNA damage, cell migration inhibition and lysosomal integrity loss in colon adenocarcinoma (HT-29) (155). Together, these studies demonstrate that pumpkin-derived bioactives exert multi-targeted anticancer, chemo preventive and pro-apoptotic effects in diverse tumor models.

### Prunus dulcis

*Prunus dulcis*, commonly known as almond, produces seeds rich in fixed oils, phenolic compounds, vitamins, minerals and unsaturated fatty acids like oleic and linoleic acids (156). Oleic acid, linoleic acid and palmitic acid have been shown to modulate BMP-2, beta-catenin, LGR-5, Jagged 1, Ki-67 expression; affects Wnt, Notch and BMP signaling pathways in colon cancer (156). Quercetin-3-o-rutinoside, kaempferol-3-o-rutinoside, isorhamnetin-3-o-galactoside, quercetin-3-o-galactoside and kaempferol-3-o-glucoside further caused selective inhibition of CYP17a1 lyase in Castration-resistant prostate cancer (CRPC) (157). Amygdalin downregulated PI3K-AKT-mTOR pathway and indirectly affected Ras thus displaying anti-tumor and anti-proliferative effects (158). Together, these findings highlight the multi-targeted anti-cancer potential of almonds through modulation of signaling pathways and enzyme activity.

### Cyamopsis tetragonoloba

Cyamopsis Tetragonoloba is commonly known as cluster bean and contains flavonoids and isoflavonoids such as daidzein, genistein and quercetin, which have anti-cancer properties. Flavonoid-enriched fractions (FEFs) have cytotoxic and anti-proliferative effects through induction of apoptosis, reduced cell viability and apoptosis or necrosis in hepatocellular carcinoma (Huh7) cells (159).

### Anisomeles malabarica

*Anisomeles Malabarica*, commonly known as pei viratti, contains active components such as anisomelic acid, quinones, flavonoids, phenols, terpenoids and beta-sitosterol. Anisomeles malabarica has anti-tumor, cytotoxic, apoptotic and anti-proliferative effects in various cancer models (160). Anisomelic acid isolated from the aerial parts of the herb was found effective in inducing DNA strand breaks and apoptosis in breast and cervical cancer cells (MCF-7, MDA-MB-231, SiHa, ME-180) (160). In a related study, it was found that n-hexane and chloroform extracts of the whole plant and Phytochemicals like ovatodiolide and citral induced apoptosis through mitochondrial membrane depolarization, DNA fragmentation, and cell cycle arrest at S and G2/M phases in HPV16-positive cervical cancer cells (161). The aqueous lead extract of the herb demonstrated cytotoxic effects on HepG2 liver cancer cells, observing a concentration-dependent inhibition and apoptosis induction (162).

### Tridax procumbens

*Tridax procumbens*, commonly known as Vettukaya Poondu, contains Phytochemicals which includes flavonoids, terpenoids, essential oils, saponins, and other secondary metabolites many of which are associated potential anticancer benefits, including cytotoxic, anti-tumor, anti-proliferative, and apoptotic effects (163).The computational in-silico study conducted by Shradha et al. investigated the active component of Tridax procumbens, luteolin, as a potential anticancer agent, demonstrating its binding capacity with MCM7 protein and predicting its anti-tumor, cytotoxic and anti-proliferative activities (164). Another study focused on green synthesis of silver nanoparticles using Tridax procumbens plant extract, showing cytotoxic and anti-proliferative action of polyphenols and peptides and their effects on A549 lung cancer cells (165). Tridax procumbens and Curcuma longa powders in combination showed synergistic cytotoxic and anti-tumor effects on A549 lung cancer cells (166). An *in vitro* study found that methanolic crude extracts and fractions exhibited cytotoxic and antioxidant effects on breast, lung, colon and leukemia cell lines (167).

### Cuminum cyminum

Cuminum cyminum, commonly known as Cumin and Seeragam, contains active components such as cuminaldehyde, cymene, and other terpenes showing anti-tumor, anti-proliferative, chemopreventive and apoptotic effects (168). An *in vitro* study using SAS cell lines showed anti-proliferative and apoptotic effects of nano-emulsion of the essential oil of Cuminum cyminum (169). Another study found that the hexane extract of cumin seeds showed cytotoxic, apoptotic and anti-proliferative effects on MG63 bone cancer cells (170). An in-silico study with a molecular modeling approach showed apigetrin, cyanaroside, and cuminum compounds might inhibit CDK8 and PR receptors, indicating potential anti-tumor, anti-proliferative and enzyme inhibitory effects (171). Further, a study demonstrated chemopreventive and anti-proliferative actions through dietary administration of ethanolic extract of cumin powder by reversing miRNA-mediated oncogenic pathways and modulating CYP1A1 (172). These findings suggest the promising anticancer potential through modulation of oncogenic pathways.

### Trigonella foenum graecum

*Trigonella foenum graecum*, commonly known as Fenugreek, is known for active components like diosgenin, saponins, polyphenols and various flavonoids, exhibiting effects like anti-tumor, cytotoxic, apoptotic, anti-proliferative and antioxidant properties (173). An *in vitro* study showed diosgenins’ anti-tumor and apoptotic effects in PC-3 prostate cancer via downregulation of NEDD4 and modulation of pAkt, P73 and LATS1. 316 Another study demonstrated anti-angiogenic and cytotoxic actions and inhibition of endothelial cell viability, tube formation, and neovascularization, potential suppression of VEGF and NF-κB signaling pathways by using ethanolic extract of fenugreek seeds (174). Fenugreek showed potential anti-tumor effects in breast cancer by high binding affinity of galactomannan to breast cancer protein (PDB ID: 3EQM), forming multiple hydrogen bonds and a stable molecular dynamics profile (175). Protein hydrolysates from fenugreek seeds showed anti-proliferative and apoptotic effects on Caco2/TC7 colon cancer cells via the G1 phase arrest and Caspase-3 activation (176). Mahmoud et al. reported that fenugreek induced apoptosis in hepatocellular carcinoma cell line HepG2 mediated by upregulation of p53 and PCNA, with anti-tumor effects (177). The active component of fenugreek, diosgenin, downregulates NEDD4 and induces apoptosis in PC-3 prostate cancer cells through pAkt suppression and p73 activation (178). Al Asmari et al. reported cytotoxicity in HepG2 and MCF-7, via apoptosis induction and caspase activation by aqueous extract of fenugreek (179). Active components like saponins in fenugreek induced apoptosis in colorectal cancer cells via ROS generation and caspase activation (180). In sum, these primary studies indicate that fenugreek-derived compounds can inhibit cancer cell viability, angiogenesis and proliferation while promoting apoptotic pathways (often via caspases and tumor-suppressor signaling) in diverse cancer types.

### Solanum nigrum

*Solanum nigrum* (black nightshade) is a medicinal herb rich in diverse bioactive compounds – notably steroidal saponins and alkaloids (e.g. solanine, solasonine, solamargine) as well as flavonoids, polyphenols, glycoproteins and polysaccharides (181). Unripe berries in particular contain high levels of these glycosides, which underlie the plant’s pharmacological potency. Extracts of S. nigrum (aqueous, ethanolic, etc.) from whole plant, leaves or fruits have demonstrated broad anticancer activity: they inhibit proliferation of many cancer cell lines (e.g. liver HepG2, cervical HeLa, breast MCF-7, ovarian, prostate, etc.) and induce apoptosis via mitochondrial and death-receptor pathways. Mechanistically, S. nigrum treatments upregulate pro-apoptotic factors (Bax, cleaved caspases, p21) and downregulate anti-apoptotic/cell-cycle proteins (Bcl-2, cyclin B1, CDK1), often through increases in ROS and caspase-9/3 activation (181, 182). The extracts also disrupt tumor-promoting signaling (e.g. AKT, STAT3, NF-κB, MAPK, VEGF/VEGFR) and metastasis-related factors (e.g. MMPs, E-cadherin) and can synergize with chemotherapies (cisplatin, doxorubicin, docetaxel) to enhance cancer cell killing (181, 182). In summary, S. nigrum’s rich mix of glycoalkaloids, saponins and polyphenols confers multi-targeted anticancer, anti-inflammatory and immunoregulatory properties, making it a promising source of therapeutic agents (181, 182).

### Cucumis sativus

Cucumis sativus, or cucumber, shows promising therapeutic potential against prostate carcinoma and benign prostatic hyperplasia (BPH) (183).In studies with Wistar rats, raw cucumber seeds and oil treatment over 28 days led to significant improvements in prostate health, including reduced prostate weight and volume, decreased total protein levels, and lower PSA which inhibit prostate hyperplasia through the inhibition of 5α-reductase (184). Additionally, C. sativus exhibits analgesic, antioxidant, antibacterial, anti-inflammatory, anti-androgenic, weak estrogenic activities, antiproliferative, anticancer properties and cytotoxic, resulting in growth arrest and apoptosis in various cancer types (183–185). Further analysis demonstrated that CuS-CuC significantly affects cell cycle regulators and apoptosis mediators in prostate, bladder, and HepG2 cells, reducing colony formation (184). In LNCaP cells, there was an accumulation of cleaved caspase-3, and in HepG2 cells, increased cleaved caspase-9 levels indicated apoptosis, while Bcl-2 levels remained steady (184). CuC was found to inhibit Akt signaling by blocking phosphorylation at Ser473, inducing apoptosis without necrosis in breast cancer cells (184). Cucumis sativus methanol extract (CSME) treatment in breast cancer cells led to significant morphological changes, including cell shrinkage, blebbing, and reduced cell population compared to untreated cells (185). The study also investigated the impact of C. sativus seed oil in combination with estrogen and letrozole prostatic cancer cell lines.

### Piper betel leaves

Piper betel, or betel, contains various bioactive compounds, including chavicol, cineol, eugenol, alkaloids, flavonoids, steroids, saponins, chavibetol, chavibetol acetate, caryophyllene, allyl pyrocatechol diacetate, camphene, chavibetol methyl ether, eugenol, a-pinene, f-pinene, u-Limonene, saprobe, 1–8- cineol, and allyl pyrocatechol monoacetate and tannins, along with sugars and essential oils (186–189). *In vitro*, hydroxychavicol‐enriched extracts induce cell-cycle arrest and apoptosis in diverse cancer cells: for example, treatment of human prostate cancer cells caused G_1_‐phase accumulation, loss of mitochondrial membrane potential, ROS overproduction and activation of caspase-3/PARP (190).These insults activate stress pathways (JNK/MAPK) and DNA damage responses leading to caspase-dependent apoptosis, while concomitantly suppressing epithelial–mesenchymal transition (EMT) and migration (190, 191). By rebalancing redox homeostasis, betel phenolics both prevent oxidative DNA damage and trigger ROS‐driven cancer cell death; notably, increased MnSOD activity induced by these compounds can suppress NF-κB/AP-1 signaling in tumor cells (192). It has a cytotoxicity and genotoxicity range from 34.91 mg/ml to 101.79 mg/ml (188). The essential oils of P. betel exhibit higher toxicity compared to crude extracts on leukocytes (188). P. betel exhibits anticancer, anti-allergic, antimicrobial, anti-platelet, and immunomodulatory actions, with potential applications against breast cancer, colon cancer, and human cervical cell lines (186–188).

### Withania somnifera

*Withania somnifera* (WS), popularly called as Ashwagandha or Indian ginseng possesses bioactive components like Withaferin A (193, 194), withanone (193), L-asparginase (195), withanolide D, withanolide O, Kaempferol (196). Withaferin A and Withanone from ashwagandha leaves displayed anti-inflammatory, anticancer, apoptotic and cytotoxic effects by binding to and preventing the homodimerization of Survivin in several cancer cell lines, including Human normal lung fibroblasts (MRC-5, TIG-3, and WI-38) and a variety of cancer cells, including colon cancer (HCT116), breast cancer (MDA-MB-231, MCF-7 and T-47D), fibrosarcoma (HT1080), non-small lung cancer (A549), cervical cancer (HeLa, ME-180, SKG-II, and CaSki), osteosarcoma (U2OS and Saos-2), and melanoma (G361) (193). Decrease in C6 glioma cells in dose dependent manner was reported in an *in vitro* study when cells were treated with doses ranging 50, 100, 200 and 500 μg/mL with downregulation of Bcl-2, upregulation of Bax, and increased expression of apoptotic markers Caspase-3 and -9 (197). A preclinical study of oral carcinoma cell lines, Ca9-22, HSC-2, HSC-3, HSC-4, demonstrated that Kaempferol and withanolide D kaempferol and withanolide D effectively inhibited oncogenic proteins, CDK2, and BRD3, inducing cytotoxicity via autophagy and caspase activation (196). L-asparaginase derived from the leaves, unripe, and ripe fruits of WS selectively deaminates asparagine, promoting cytotoxicity in acute and chronic lymphoblastic leukemia (195).

## Discussion

**Figure 2:**
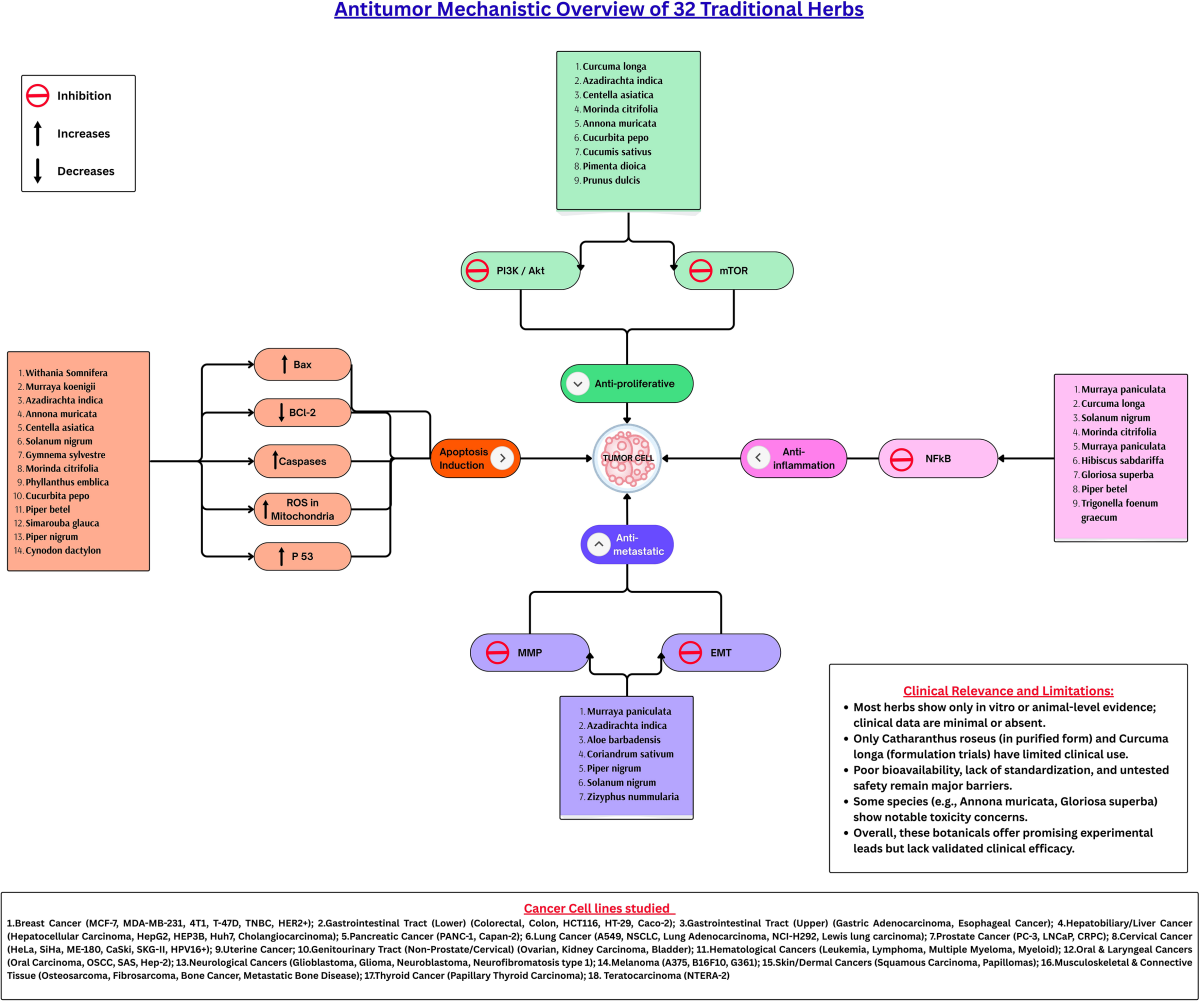
Antitumor mechanistic overview of 32 traditional herbs. The 32 traditional medicinal herbs exert their antitumor effects through multiple converging pathways. These herbs modulate four principal mechanisms: induction of apoptosis, suppression of proliferation, inhibition of inflammation, and blockade of metastasis. Apoptosis is facilitated by upregulating Bax, p53, and caspases while downregulating Bcl-2 and enhancing mitochondrial ROS activity. The anti-proliferative effect is primarily achieved through inhibition of the PI3K/Akt/mTOR axis, whereas NF-κB suppression mediates the anti-inflammatory response. Concurrently, inhibition of MMPs and reversal of EMT signaling prevent cancer cell migration and invasion. Collectively, these pathways underscore the multi-targeted and synergistic potential of phytochemicals in regulating tumor progression. Bax, Bcl-2-associated X protein; Bcl-2, B-cell lymphoma 2; p53, Tumor Protein p53; ROS, Reactive Oxygen Species; PI3K, Phosphoinositide 3-kinase; Akt, Protein Kinase B; mTOR, Mechanistic Target of Rapamycin; NF-κB, Nuclear Factor kappa-light-chain-enhancer of activated B cells; MMPs, Matrix Metalloproteinases; EMT, Epithelial-Mesenchymal Transition.

This narrative review provides an integrative analysis of 32 herbs with claimed anticancer properties, rooted in traditional use and ethnobotanical knowledge from Tamil Nadu, India. By synthesizing data from peer-reviewed literatures, the review highlights both the depth and limitations of existing evidence supporting these herbs. **Figure 2** provides an overview of the key mechanisms underlying the anticancer potential of the herbs discussed in this study.

Herbs with reported anticancer effects show differences in the depth and quality of evidence. For example, *Withania somnifera*, *Curcuma longa*, and *Annona muricata*, have been extensively studied *in vitro* and in animal models with identified mechanisms (apoptosis induction, angiogenesis inhibition, anti-metastatic effects, and immune modulation (4, 198, 199). By contrast, many other traditionally used herbs have virtually no preclinical animal or clinical data. This disparity highlights critical gaps, some high-use plants lack validation. This discrepancy underscores a critical research gap several high-use herbs in folk medicine continue to lack systematic validation of their anticancer efficacy or safety.

Several herbs identified in this review appear to be promising candidates for future clinical research and potential therapeutic application. *Withania somnifera* contains withanolides that show broad anti-cancer activity and immunomodulatory effects. Its safety record is favorable in small trials, and it alleviated chemotherapy-induced fatigue (200). *Curcuma longa*’s curcuminoids modulate multiple cancer pathways (NF-κB, STAT3, PI3K/Akt etc.) and enhance immune responses. Curcumin is well-tolerated in humans (numerous Phase I/II trials) though its low bioavailability is a hurdle (201). Other promising candidates include *Morinda citrifolia* with Phase I data showing no dose-limiting toxicity (202).

By contrast, *Annona muricata* despite potent anticancer acetogenins must be approached cautiously its acetogenin annonacin is a potent mitochondrial toxin linked to Parkinsonian neurodegeneration (203).Likewise, the vinca alkaloids from *Catharanthus roseus* are clinically active but highly toxic (peripheral neuropathy and marrow suppression) (204), underscoring that efficacy must be balanced with safety. Further, systematic validation through controlled *in vivo* and clinical studies will be essential to distinguish promising candidates from those posing unacceptable risks, ultimately guiding their responsible integration into evidence-based cancer care.

Studies suggest that some herbal compounds can potentiate conventional cancer therapies. Curcumin, for instance, enhances chemotherapy efficacy its combination with cisplatin in papillary thyroid cancer cells produced stronger STAT3 inhibition and apoptosis than either treatment alone (201). Likewise, *Withania somnifera’s* active constituent withaferin A increases radio and chemosensitivity, showing synergistic effects with sorafenib through apoptosis induction and suppression of oncogenic signaling (200). Despite these promising findings, clinical translation remains limited, as large randomized trials integrating such herbs with standard cancer therapies are still lacking.

### Safety

A critical gap in the current evidence is safety. Many studies overlook toxicity or use non-standardized extracts. For instance, *Annona muricata* preparations vary widely in acetogenin content, and chronic exposure is neurotoxic (203). *Catharanthus roseus* derivatives vincristine/vinblastine are clinically potent but have neuropathy and myelosuppression (204).Curcumin is generally safe at dietary levels, but recent reports of liver toxicity from adulterated supplements highlight regulatory risks (201).For most herbs, human safety data are non-existent. Therefore, regulatory authorities should mandate rigorous phytochemical characterization and adherence to good manufacturing practices, even for traditional remedies. This review emphasizes that traditional use does not equate to proven safety, and scientific validation is essential before clinical application.

From a policy perspective, preserving traditional plant knowledge and supporting collaborative research is essential. Regulatory frameworks should ensure intellectual property protection, quality control, and ethical study designs. Multidisciplinary teams including oncologists, pharmacognosists, ethnobotanists, and AYUSH practitioners can accelerate translation of traditional remedies into evidence-based integrative oncology. The findings from this review aligns with the earlier evidence based reviews that call for integration of herbal/plant-based remedies with conventional cancer care (205).

### Limitations

This narrative review has several methodological constraints. First, it did not employ systematic criteria to assess the quality, risk of bias, or methodological rigor of the included studies, which limits the strength of inferences. Second, as a narrative synthesis, it lacks quantitative synthesis or meta-analysis, making it susceptible to subjective interpretation. Third, many included studies rely on preclinical models, small pilot trials, or non-standardized herbal preparations, which reduces generalizability and reproducibility. Finally, publication bias and selective reporting cannot be excluded, further constraining the certainty of the results discussed. Another key limitation of this review is the potential selection bias arising from the inclusion of herbs based on anecdotal evidence, popular traditional use, and reports from herbal activists, rather than through a systematic ethnobotanical survey; however, this approach was intended to capture herbs of real-world relevance that are actively used in traditional cancer care settings or popularly believed to be anti-cancerous. Future studies should adopt a systematic ethnobotanical or participatory rural appraisal framework to minimize potential bias and enhance reproducibility.

## Conclusion

This review identifies several promising herbal candidates with anticancer and immunomodulatory potential particularly *Withania somnifera*, *Curcuma longa*, *Azadirachta indica*, and *Morinda citrifolia.* These herbs merit priority in future clinical trials due to their multi-target mechanisms and preliminary safety data. However, some plants with known or uncertain toxicity, (e.g.: *Annona muricata*), require caution. Future research should focus on standardized extracts, defined dosing, and rigorous safety monitoring, while testing potential synergies with chemotherapy and immunotherapy. Policy support is essential to enable well-designed clinical trials that respect traditional knowledge and ensure quality control. In summary, traditional herbs offer significant promise for integrative oncology, but their translation into clinical use demands robust pharmacological validation, ethical testing, and interdisciplinary collaboration bridging ethnomedicine with modern cancer science.
