# Supplementary material for: Exploring the anticancer potential of traditional herbs from Tamil Nadu: a narrative review of ethnomedicinal insights and scientific evidence
Source: Front Immunol. 2025 Nov 19;16:1680062. doi: 10.3389/fimmu.2025.1680062 (PMC12672482; doi:10.3389/fimmu.2025.1680062)
Supplement: Supplementary file 1 [file Table1.docx]

**Overview of Preclinical and Clinical Evidence of the Anticancer Potential of 32 Anecdotal Herbs**

| **Herbs** | **Cancer types & cell lines studied** | **Strength of Evidence (preclinical/clinical)** | **Clinical data/ Promising Scope for trial** | **Barriers to translation** |
| --- | --- | --- | --- | --- |
| **Annona muricata** (Graviola) | Breast(MCF7, MDA-MB-468**, MDA-MB-231, AMJ13, prostate(PC-3, PNT1-A, DU-145, BPH-1, HEP-2), colon(HCT-116, DLD-1), pancreatic(**COLO357, HPAF, Capan-1), **lung(H1299, A549), liver(HepG2, Hep2), hematological (leukemia)(K562, THP-1, head & neck(SCC-25), and cervical**cancer(HeLa).^1,2^ | Preclinical | No controlled clinical trials in cancer | Known neurotoxicity: annonacin causes dopaminergic neuron loss(parkinsonism).^3^ |
| **Simarouba glauca** (Paradise tree) | Breast (MCF7)**, Colorectal (**HCT-116, HCT-15**), Lung cancer (A549) ^4–6^** | Invitro only | No animal or human trials reported to date. | No in vivo efficacy/toxicity data |
| **Catharanthus roseus** (Periwinkle) | **Breast (MCF7), Acute leukemia (ALL/AML) (K562), Hodgkin’s disease, non-Hodgkin’s lymphoma^7,8^** | (Clinically approved chemotherapeutics.) | Used clinically in purified form for leukemia/lymphoma treatment.^9^ | Severe dose-limiting toxicities: peripheral neuropathy and myelosuppression are common.^9^ |
| **Azadirachta indica** (Neem) | Breast (MCF7, MDA-MB-231, SK-BR-3)**, colon, lung(A549), prostate (PC3, LNCaP), choriocarcinoma (BeWo), Lymphoma, stomach (AZ521),**cervical (HeLa) and **leukemia (U937, HL-60, THP1, B16) ^10^** | Preclinical | No clinical trials in cancer have been reported. | Safety and dosing in humans are unknown. |
| **Citrus limon** (Lemon) | Breast (MDA-MB-231, MCF-7, MDA-MB-435), hepatocellular (HepG2), leukemia (NALM-6, U937) Colon (HT-29, HCT116), lung (A549), Prostrate Cancer ^11,12^ | Preclinical only | None | Safety and dosing in humans are unknown. |
| **Justicia gendarussa** | Breast (MDA-MB-231, MDA-MB-468, MCF7**), colon (HT-29), cervical (HeLa), and pancreatic** cancer (BxPC-3, MIAPaCa-2, Panc-1) ^13,14^ | Preclinical | No animal or clinical studies have been done. | Active constituents not isolated; extracts are unstandardized; toxicity/safety is unknown. |
| **Murraya paniculata** (Orange jasmine) | **Lung adenocarcinoma (A549),** Breast Cancer (MDA-MB-231^15,16^ | Invitro only | None |  |
| **Murraya koenigii** (Curry leaf) | Colon (HT-29), lung(A549), liver (HepG2), skin (CLS-354, pancreatic (CAPAN-2, BxPC3, CFPAC1, HPAFII, SW 1190), cervical (HeLa), prostate (PC-3), brain (U87MG, LN229), connective tissue (HL-60, K562, MOLT-3), breast (MCF-7, MDA-MB-231^17^ | Preclinical | None | No known toxicity. Safety and dosing in humans are unknown. |
| **Hibiscus sabdariffa** (Roselle) | Breast (HCC-70, MCF7, MDA-MB-231), connective tissue (MCA-205), colon (CT26), liver (HepG2)^18–20^ | Invitro only | None | Requires in vivo validation, then the human trials |
| **Centella asiatica** (Gotu kola) | Liver (HepG2), Oral squamous carcinoma^21,22^ | Preclinical | None | Poor pharmacokinetics: Asiatic acid has low solubility and bioavailability.^23^ |
| **Morinda centifolia** (Noni) | Breast cancer (MDA-MB-231, MCF7), Lung cancer (A549, LL2), General cancer pathogenesis (K-ras^ts^-NRK, JB6), prostate (PC-3), Oral (H103, H400, H413, H357, H376, H314), Eye (Y79) Cervical cancer (HeLa, SiHa), Leukemia (jurkat cells), liver (Hep G2), Skin (MUM-2B) Colon cancer (HCT116, HT-29, SW480, LoVo)^24^ | Preclinical & some clinical | One Phase I trial (advanced cancer, found no acute toxicity).^25^ No RCTs completed. | Translational gaps: Only anecdotal/phase I evidence; no large trials.^25^ Raw material consistency and funding are obstacles. |
| **Pimenta dioica** (Allspice) | Breast cancer (MDA-MB231)^26^ | Preclinical | None | Requires in vivo validation, then the human trials |
| **Cynodon dactylon**(Bermuda grass) | Hepatic Cancer, Colon cancer (COLO 320 DM, MCH-7, AGS, A549)^27,28^ | Preclinical | None | Toxicity profile not well studied; translation awaits further research. |
| **Zizyphus nummularia** | Breast, leukemia, ovarian, colon, kidney, connective tissue (S-180), pancreatic (CAPAN-2), cervical cancers (HeLa).^29^ | Preclinical | None | Lack of pharmacological and clinical studies. |
| **Curcuma longa** (Turmeric) | Breast (MCF-7, MCF-10A, BT-483, MDA-MB-231), colon (HT-29, CT-26), pancreatic, leukemia (KBM-5, type 562), prostate (PC3, 22Rv1, TRAMP-C1, LNCaP, DU145, C4-2B), lung (A549), liver (HepG2), brain (U251MG), ovarian (SKV3), head and neck (CAL27, UM-SCC1), melanoma (B16F10, A385) cancers^30^ | Preclinical | Multiple Phase I/II trials (mostly using curcuminoid formulations) report good tolerability but no proven efficacy yet.^31^ | Poor absorption and rapid metabolism of curcumin. Reports of hepatotoxicity from adulterated supplements.^31^ |
| **Aloe barbadense** (Aloe vera) | Breast cancer (MCF-7), cervical cancer (HeLa), neuroblastoma (SH-SY5Y), prostate cancer, bladder cancer, pancreatic cancer, non-small-cell lung cancer, and hepatocellular carcinoma (HepG2).^32–34^ | Preclinical | No controlled cancer trials; tested (ineffectively) for chemo-mucositis | Toxicity profile not well studied; translation awaits further research. |
| **Gymnema sylvestre** | Cervical cancer (HeLa), lung cancer (A549), breast cancer (MCF7), melanoma (A375), leukaemia (myeloid), gastric cancer, and skin papilloma^35^ | Preclinical | None | Lack of human data; unknown pharmacokinetics |
| **Gloriosa superba** | Lung (A549 NCI-H460, HOP-62), colon (COLO 205, HCT 116), breast (MCF7, MDA-MB-231, Hs 578T), leukemia (MOLT-4, HL-60), cervix (SiHa), oral (FaDu, SCC-9), brain (SF-295, U251) ^36^ | Preclinical | None | Very high toxicity: whole plant is poisonous (colchicine causes fatal effects).^37^ |
| **Piper nigrum** (Black pepper) | Breast (MCF-7, MDA-MB-231), ovarian (A2780, OVCAR3), gastric (TMK1), glioma (U251-MG), lung (A549, H441), cervical (HeLa/MMC), oral (KB cell line) rectal (HRT-18, HT29, DLD-1), prostate (PC-3, DU-145, LNCaP, 22RV1 PCa), leukemia (HL60), etc.^38^ | Preclinical | No human trials | Phytochemical variability and poor bioavailability.^39^ |
| **Coriandrum sativum** (Coriander) | Hepatocellular carcinoma (HepG2), Melanoma (B16F10),^40^ Breast (MCF7)^41^, Prostrate cancer (PC3, LNCaP)^42^ | Preclinical | None | Active compound(s) unidentified |
| **Phyllanthus emblica**(Gooseberry) | Colon (SW620), breast (MCF10A, MDA-MB-231, MDA-MB-435, and MDA-MB-468), liver (HepG2, Hs578T), lung (A549), cervical (HeLa), ovarian (SK-OV3), gastric^43,44^ | Preclinical | None | Human effects unknown |
| **Cucurbita pepo** (Pumpkin) | Melanoma (A375), gastric cancer (NUGC-3, SGC7901/DDP,breast cancer (MCF-7, MDA-MB-231, SKBR-3, 4T1), prostate cancer (PC-3, LNCaP,ovarian cancer (HEY, SKOV3), and colon cancer (SW480). ^45^ | Preclinical | None | Lack of human data, unknown pharmacokinetics for cucurbitacin compounds |
| **Prunus dulcis** (Almond) | Lung (A549), ovarian (SKOV3), prostate (PC3), leukemia (NB4, U937), liver (Huh7, Hep-2), bladder (T-24) cancer^46^ | Preclinical | None | Human effects unknown |
| **Cyamopsis tetragonoloba** (Guar) | Liver Cancer (Huh-7)^47^ | In vitro | None | Toxicity and safety profiles not yet evaluated. |
| **Anisomeles malabarica** | Cervical cancer (SiHa, ME 180)^48^ | In vitro | None | Safety unknown; no pharmacological profiling in animals. |
| **Tridax procumbens** | Lung (A549), breast (MCF-7), liver (HepG2), squamous carcinoma (A431), breast carcinoma (MDA-MB-231), breast adenocarcinoma (MDA-MB 468), colon carcinoma (COLO-205), erythroleukemia (K562), prostate (PC3) cancer cells^49,50^ | In vitro | None | Toxicity and safety profiles not yet evaluated. |
| **Cuminum cyminum** (Cumin) | Bone cancer (MG63), Colon Cancer (SW480) ^51,52^ | Preclinical | None | Active ingredient(s) not standardized; clinical efficacy untested. |
| **Trigonella foenum-graecum** (Fenugreek) | Prostate, breast (MCF-7), colon (HT-29), lung (A549), liver, skin, thyroid, pancreatic, blood (leukemia and lymphoma)^53^ | In vitro | None | Efficacy unproven in vivo; mechanisms need elucidation. |
| **Solanum nigrum** (Black nightshade) | Breast (4T1), prostate (DU145, PC3), lungs (A549, H1650, H1975, PC9, H1299), ovarian, cholangiocarcinoma (QBC939), oesophagal (EC9706, KYSE30, Eca109), colorectal (SW480, SW620, HT-29, RKO, HCT 116), Osteosarcoma (HOS, U20S), gastric (SGC-7901, SNU1, SNU5, NCI-N87, HGC27), pancreatic (SW1990, PANC1), liver cancers (HepG2, SMMC7721, LO2, QGY7703, H22, Hep3b)[.^54^](https://pmc.ncbi.nlm.nih.gov/articles/PMC10773844/#:~:text=Furthermore%2C%20the%20aqueous%20extract%20can,26) | In vitro | None | No clinical data. |
| **Cucumis sativus** (Cucumber) | Breast (MCF-7), cervical (HeLa), and prostate cancers.^55,56^ | In vitro | None | No clinical data. |
| **Piper betel** (Betel leaf) | Prostate cancer, Breast Cancer(MCF7) ^57,58^ | Preclinical | None | Human safety unclear despite bioactivity. |
| **Withania somnifera**(Ashwagandha) | Breast cancer (MCF-7), colon, lung, prostate and blood cancer^59^ | Preclinical | None | No clinical data, Optimal dosing/formulation unclear. |

**References**

1. Pathirana OC, Paranagama MP, Wijesundera KK, Mahakapuge TAN, Abeykoon AMAU, Rajapakse J. Elucidating the potential of Annona muricata L. grown in Sri Lanka to be used in developing an anticancer drug against colorectal and breast cancers. *BMC Complement Med Ther*. 2024;24(1):1-16. doi:10.1186/S12906-024-04712-X/FIGURES/11

2. Ilango S, Sahoo DK, Paital B, et al. A Review on Annona muricata and Its Anticancer Activity. *Cancers (Basel)*. 2022;14(18):4539. doi:10.3390/CANCERS14184539

3. Champy P, Höglinger GU, Féger J, et al. Annonacin, a lipophilic inhibitor of mitochondrial complex I, induces nigral and striatal neurodegeneration in rats: possible relevance for atypical parkinsonism in Guadeloupe. *J Neurochem*. 2004;88(1):63-69. doi:10.1046/J.1471-4159.2003.02138.X

4. Ramasamy SP, Rajendran A, Pallikondaperumal M, et al. Broad-Spectrum Antimicrobial, Antioxidant, and Anticancer Studies of Leaf Extract of Simarouba glauca DC In Vitro. *Antibiotics*. 2022;11(1):59. doi:10.3390/ANTIBIOTICS11010059

5. Gurudhathan KB, Peerzada J, Prakesh A, Mohamed Jaabir MS. Exploring the anti-cancer potential of methanolic extract from Simarouba glauca: Induction of apoptosis and growth inhibition in lung cancer cells. *Oral Oncology Reports*. 2023;8:100104. doi:10.1016/J.OOR.2023.100104

6. Jose A, Chaitanya MVNL, Kannan E, Madhunapantula SR V. Tricaproin isolated from Simarouba glauca inhibits the growth of human colorectal Carcinoma cell lines by targeting Class-1 Histone deacetylases. *Front Pharmacol*. 2018;9(MAR):127. doi:10.3389/FPHAR.2018.00127/FULL

7. Vincristine: Uses, Interactions, Mechanism of Action | DrugBank Online. Accessed July 29, 2025. https://go.drugbank.com/drugs/DB00541

8. Goswami S, Ali A, Prasad ME, Singh P. Pharmacological significance of Catharanthus roseus in cancer management: A review. *Pharmacological Research - Modern Chinese Medicine*. 2024;11:100444. doi:10.1016/J.PRMCM.2024.100444

9. Chen RJ, Arora RD, Menezes RG. Vinca Alkaloid Toxicity. *StatPearls*. Published online March 10, 2024. Accessed October 22, 2025. https://www.ncbi.nlm.nih.gov/books/NBK557842/

10. Hao F, Kumar S, Yadav N, Chandra D. Neem components as potential agents for cancer prevention and treatment. *Biochim Biophys Acta*. 2014;1846(1):247. doi:10.1016/J.BBCAN.2014.07.002

11. Wang L, Wang J, Fang L, et al. Anticancer Activities of Citrus Peel Polymethoxyflavones Related to Angiogenesis and Others. *Biomed Res Int*. 2014;2014:453972. doi:10.1155/2014/453972

12. Ezzat RS, Abdel-Moneim A, Zoheir KM, et al. Anti-carcinogenic effects and mechanisms of actions of Citrus limon fruit peel hydroethanolic extract and limonene in diethylnitrosmine/2-acetylaminofluorene-induced hepatocellular carcinoma in Wistar rats. *Am J Cancer Res*. 2024;14(11):5193. doi:10.62347/FOYI6658

13. Ayob Z, Mohd Bohari SP, Abd Samad A, Jamil S. Cytotoxic Activities against Breast Cancer Cells of Local Justicia gendarussa Crude Extracts. *Evid Based Complement Alternat Med*. 2014;2014:732980. doi:10.1155/2014/732980

14. Ayob Z, Mohd Bohari SP, Abd Samad A, Jamil S. Cytotoxic Activities against Breast Cancer Cells of Local Justicia gendarussa Crude Extracts. *Evid Based Complement Alternat Med*. 2014;2014:732980. doi:10.1155/2014/732980

15. He SD, Yang XT, Yan CC, et al. Promising Compounds From Murraya exotica for Cancer Metastasis Chemoprevention. *Integr Cancer Ther*. 2016;16(4):556. doi:10.1177/1534735416678981

16. Joshi D, Gohil KJ. A Brief Review on Murraya paniculata (Orange Jasmine): pharmacognosy, phytochemistry and ethanomedicinal uses. *J Pharmacopuncture*. 2023;26(1):10. doi:10.3831/KPI.2023.26.1.10

17. Aniqa A, Kaur S, Sadwal S. A Review of the Anti-Cancer Potential of Murraya koenigii (Curry Tree) and Its Active Constituents. *Nutr Cancer*. 2022;74(1):12-26. doi:10.1080/01635581.2021.1882509

18. Yasmin R, Gogoi S, Bora J, et al. Novel Insight into the Cellular and Molecular Signalling Pathways on Cancer Preventing Effects of Hibiscus sabdariffa: A Review. *J Cancer Prev*. 2023;28(3):77. doi:10.15430/JCP.2023.28.3.77

19. Ezcurra-Hualde M, Gómez-Leyva JF, Juarez-Curiel E, et al. Intratumoral administration of Hibiscus sabdariffa-derived anthocyanins exerts potent antitumor effects in murine cancer models. *Front Immunol*. 2025;16:1549890. doi:10.3389/FIMMU.2025.1549890/BIBTEX

20. Millan ZE, García-Garcia MR, Leyva JG, et al. Lyophilized Extract of Hibiscus sabdariffa L. Induces Cytotoxicity in Breast Cancer Cell Lines. *Proceedings 2020, Vol 61, Page 22*. 2020;61(1):22. doi:10.3390/IECN2020-07002

21. Vijayakumar T, Rameshkumar A, Krishnan R, Bose D, Vasanthi V, Nandhini G. Evaluation of the Anti-Carcinogenic Effect of Centella Asiatica on Oral Cancer Cell Line: In vitro Study. *Asian Pac J Cancer Prev*. 2023;24(5):1695. doi:10.31557/APJCP.2023.24.5.1695

22. Hussin F, Eshkoor SA, Rahmat A, Othman F, Akim A. The centella asiatica juice effects on DNA damage, apoptosis and gene expression in hepatocellular carcinoma (HCC). *BMC Complement Altern Med*. 2014;14(1):1-7. doi:10.1186/1472-6882-14-32/TABLES/1

23. Chen R, Zhang W, Zhang M, Liu W, Feng W, Zhang Y. Asiatic acid in anticancer effects: emerging roles and mechanisms. *Front Pharmacol*. 2025;16:1545654. doi:10.3389/FPHAR.2025.1545654/BIBTEX

24. Chanthira Kumar H, Lim XY, Mohkiar FH, Suhaimi SN, Mohammad Shafie N, Chin Tan TY. Efficacy and Safety of Morinda citrifolia L. (Noni) as a Potential Anticancer Agent. *Integr Cancer Ther*. 2022;21:15347354221132848. doi:10.1177/15347354221132848

25. Issell BF, Gotay CC, Pagano I, Franke AA. Using quality of life measures in a Phase I clinical trial of noni in patients with advanced cancer to select a Phase II dose. *J Diet Suppl*. 2009;6(4):347-359. doi:10.3109/19390210903280272

26. Zhang L, Shamaladevi N, Jayaprakasha G, Patil BS, Lokeshwar BL. Polyphenol-rich extract of Pimenta dioica berries (Allspice) kills breast cancer cells by autophagy and delays growth of triple negative breast cancer in athymic mice. *Oncotarget*. 2015;6(18):16379. doi:10.18632/ONCOTARGET.3834

27. Albert-Baskar A, Ignacimuthu S. Chemopreventive effect of Cynodon dactylon (L.) Pers. extract against DMH-induced colon carcinogenesis in experimental animals. *Experimental and Toxicologic Pathology*. 2010;62(4):423-431. doi:10.1016/J.ETP.2009.06.003

28. Kowsalya R, Kaliaperumal J, Vaishnavi M, Namasivayam E. Anticancer activity of Cynodon dactylon L. root extract against diethyl nitrosamine induced hepatic carcinoma. *South Asian J Cancer*. 2015;4(2):83. doi:10.4103/2278-330X.155691

29. Mesmar J, Abdallah R, Badran A, Maresca M, Shaito A, Baydoun E. Ziziphus nummularia: A Comprehensive Review of Its Phytochemical Constituents and Pharmacological Properties. *Molecules 2022, Vol 27, Page 4240*. 2022;27(13):4240. doi:10.3390/MOLECULES27134240

30. Tomeh MA, Hadianamrei R, Zhao X. A Review of Curcumin and Its Derivatives as Anticancer Agents. *International Journal of Molecular Sciences 2019, Vol 20, Page 1033*. 2019;20(5):1033. doi:10.3390/IJMS20051033

31. PDQ Integrative, Alternative, and Complementary Therapies Editorial Board. Curcumin (Curcuma, Turmeric) and Cancer (PDQ®): Health Professional Version. *PDQ Cancer Information Summaries*. Published online 2002. Accessed October 22, 2025. https://www.cancer.gov/about-cancer/treatment/cam/hp/curcumin-pdq

32. Shalabi M, Khilo K, Zakaria MM, Elsebaei MG, Abdo W, Awadin W. Anticancer activity of Aloe vera and Calligonum comosum extracts separetely on hepatocellular carcinoma cells. *Asian Pac J Trop Biomed*. 2015;5(5):375-381. doi:10.1016/S2221-1691(15)30372-5

33. Zimbone S, Romanucci V, Zarrelli A, et al. Exploring the therapeutic potential of Aloin: unraveling neuroprotective and anticancer mechanisms, and strategies for enhanced stability and delivery. *Scientific Reports 2024 14:1*. 2024;14(1):1-12. doi:10.1038/s41598-024-67397-9

34. Hussain A, Sharma C, Khan S, Shah K, Haque S. Aloe vera inhibits proliferation of human breast and cervical cancer cells and acts synergistically with cisplatin. *Asian Pacific Journal of Cancer Prevention*. 2015;16(7):2939-2946. doi:10.7314/APJCP.2015.16.7.2939,

35. Khan F, Sarker MMR, Ming LC, et al. Comprehensive review on phytochemicals, pharmacological and clinical potentials of gymnema sylvestre. *Front Pharmacol*. 2019;10(OCT):474326. doi:10.3389/FPHAR.2019.01223/XML

36. Goel B, Dey B, Chatterjee E, et al. Antiproliferative Potential of Gloriosine: A Lead for Anticancer Drug Development. *ACS Omega*. 2022;7(33):28994-29001. doi:10.1021/ACSOMEGA.2C02688/SUPPL_FILE/AO2C02688_SI_001.PDF

37. Joshi BC, Durgapal S, Mukhija M, Bhargava A. An overview on the phytopharmacological insights into Gloriosa superba L. (Kalahari): a promising endangered plant species. *Discover Plants 2024 1:1*. 2024;1(1):1-18. doi:10.1007/S44372-024-00054-7

38. Mitra S, Anand U, Jha NK, et al. Anticancer Applications and Pharmacological Properties of Piperidine and Piperine: A Comprehensive Review on Molecular Mechanisms and Therapeutic Perspectives. *Front Pharmacol*. 2022;12:772418. doi:10.3389/FPHAR.2021.772418/XML

39. Wiraswati HL, Ma’ruf IF, Hidayati NA, Ramadhanti J, Calina D, Sharifi-Rad J. Harnessing the anticancer potential of Piper nigrum: a synergistic approach to chemotherapy enhancement and reduced side effects. *Discover Oncology*. 2025;16(1):10. doi:10.1007/S12672-024-01716-4

40. Huang H, Nakamura T, Yasuzawa T, Ueshima S. Effects of coriandrum sativum on migration and invasion abilities of cancer cells. *J Nutr Sci Vitaminol (Tokyo)*. 2020;66(5):468-477. doi:10.3177/JNSV.66.468,

41. Tang ELH, Rajarajeswaran J, Fung SY, Kanthimathi MS. Antioxidant activity of Coriandrum sativum and protection against DNA damage and cancer cell migration. *BMC Complement Altern Med*. 2013;13:347. doi:10.1186/1472-6882-13-347

42. Elmas L, Secme M, Mammadov R, Fahrioglu U, Dodurga Y. The determination of the potential anticancer effects of Coriandrum sativum in PC-3 and LNCaP prostate cancer cell lines. *J Cell Biochem*. 2019;120(3):3506-3513. doi:10.1002/JCB.27625,

43. Zhao T, Sun Q, Marques M, Witcher M. Anticancer Properties of Phyllanthus emblica (Indian Gooseberry). *Oxid Med Cell Longev*. 2015;2015(1):950890. doi:10.1155/2015/950890

44. Baliga MS, Dsouza JJ. Amla (Emblica officinalis Gaertn), a wonder berry in the treatment and prevention of cancer. *European Journal of Cancer Prevention*. 2011;20(3):225-239. doi:10.1097/CEJ.0B013E32834473F4

45. Varela C, Melim C, Neves BG, et al. Cucurbitacins as potential anticancer agents: new insights on molecular mechanisms. *J Transl Med*. 2022;20(1):630. doi:10.1186/S12967-022-03828-3

46. Shalayel MHF, Al-Mazaideh GM, Alanezi AA, Almuqati AF, Alotaibi M. The Potential Anti-Cancerous Activity of Prunus amygdalus var. amara Extract. *Processes 2023, Vol 11, Page 1277*. 2023;11(4):1277. doi:10.3390/PR11041277

47. Asati V, Srivastava A, Mukherjee S, Sharma PK. Comparative analysis of antioxidant and antiproliferative activities of crude and purified flavonoid enriched fractions of pods/seeds of two desert legumes Prosopis cineraria and Cyamopsis tetragonoloba. *Heliyon*. 2021;7(6):e07304. doi:10.1016/J.HELIYON.2021.E07304

48. Preethy C, Padmapriya R, Periasamy V, et al. Antiproliferative property of n-hexane and chloroform extracts of Anisomeles malabarica (L). R. Br. in HPV16-positive human cervical cancer cells. *J Pharmacol Pharmacother*. 2012;3(1):26. doi:10.4103/0976-500X.92500

49. Sagheer R, Gupta A, Luqman S, et al. Antiproliferative and antioxidant potential of Tridax procumbens extracts against various human cancer cell lines: An insight for medicines from weeds. *J King Saud Univ Sci*. 2024;36(10). doi:10.1016/J.JKSUS.2024.103474

50. Syed A, Benit N, Alyousef AA, Alqasim A, Arshad M. In-vitro antibacterial, antioxidant potentials and cytotoxic activity of the leaves of Tridax procumbens. *Saudi J Biol Sci*. 2020;27(2):757-761. doi:10.1016/j.sjbs.2019.12.031

51. Rostami L, Soltanzadeh H, Rad FA. Anti-proliferative Effects of Cuminum cyminum Extraction by Co-administration of Layered Double Hydroxide (LDH) Nanosheets on SW480 Colorectal Cancer Cell Line through Apoptosis Induction. *Curr Cancer Ther Rev*. 2022;18(4):310-315. doi:10.2174/1573394718666220707112238/CITE/REFWORKS

52. Chandrasekaran R, Krishnan M, Chacko S, et al. Assessment of anticancer properties of cumin seed (Cuminum cyminum) against bone cancer. *Front Oncol*. 2023;13:1322875. doi:10.3389/FONC.2023.1322875/BIBTEX

53. El Bairi K, Ouzir M, Agnieszka N, Khalki L. Anticancer potential of Trigonella foenum graecum: Cellular and molecular targets. *Biomedicine & Pharmacotherapy*. 2017;90:479-491. doi:10.1016/J.BIOPHA.2017.03.071

54. Zhang H, Lv JL, Zheng QS, Li J. Active components of Solanum nigrum and their antitumor effects: a literature review. *Front Oncol*. 2023;13:1329957. doi:10.3389/FONC.2023.1329957

55. Tuama AA, Mohammed AA. Phytochemical screening and in vitro antibacterial and anticancer activities of the aqueous extract of Cucumis sativus. *Saudi J Biol Sci*. 2019;26(3):600-604. doi:10.1016/j.sjbs.2018.07.012

56. Tuama AA, Mohammed AA. Phytochemical screening and in vitro antibacterial and anticancer activities of the aqueous extract of Cucumis sativus. *Saudi J Biol Sci*. 2019;26(3):600-604. doi:10.1016/j.sjbs.2018.07.012

57. Abrahim NN, Kanthimathi MS, Abdul-Aziz A. Piper betle shows antioxidant activities, inhibits MCF-7 cell proliferation and increases activities of catalase and superoxide dismutase. *BMC Complement Altern Med*. 2012;12:220. doi:10.1186/1472-6882-12-220

58. Paranjpe R, Gundala SR, Lakshminarayana N, et al. Piper betel leaf extract: Anticancer benefits and bio-guided fractionation to identify active principles for prostate cancer management. *Carcinogenesis*. 2013;34(7):1558-1566. doi:10.1093/CARCIN/BGT066,

59. Singh N, Yadav SS, Rao AS, et al. Review on anticancerous therapeutic potential of Withania somnifera (L.) Dunal. *J Ethnopharmacol*. 2021;270. doi:10.1016/J.JEP.2020.113704,
